# A Case of Unusually Late Chloroform Asphyxia

**Published:** 1879-06

**Authors:** F. C. Hotz


### Article IV.

### A Case of Unusually Late Chloroform Asphyxia. By F. C. Hotz, m.d. Reported to the Chicago Medical Society, May 5, 1879.

Serious accidents during chloroform anæsthesia usually occur while the anæsthetic is being administered or immediately after it has been discontinued. Only when a patient has been submitted to a prolonged narcosis, accidents attributable to the action of chloroform have been known to happen sometime after its discontinuance.*

But it is very unusual that a serious accident occurs at this late period to a patient who has received a minimum dose of chloroform, just sufficient to produce a short narcosis for the performance of a short operation.

For this reason I believe the following case is remarkable and worthy of being recorded:

On the 24th of February, at 3 o'clock p. m., Thomas Jones, aged 8 years, came to the Illinois Charitable Eye and Ear Infirmary for the operation for convergent strabismus. His mother, who accompanied him, informed me that he had not eaten anything since breakfast. For at a previous visit I had told her that the boy should take no dinner on the day of the operation.

The patient, a very bright and healthy boy, was so glad to get rid of his disfigurement that he submitted without the least struggle to the administration of chloroform. His lungs and heart were normal in every respect. Chloroform was administered in an Allis' inhaler, and after about five minutes the boy was profoundly asleep. The apparatus was then removed and the operation proceeded with. The right internal rectus muscle was first tenotomized, and during this time pulse and respiration were perfectly regular. I put the speculum in the left eye, pinched up a fold of conjunctiva over the left internal rectus, and was just going to incise it with scissors when the boy began to hiccough just as though he was going to vomit. Thinking it was an effort at vomiting, I withdrew the instruments; but at the same instant the boy's face assumed a deep dusky color, respiration ceased and the pupil of the left eye, though fully exposed to the light by the speculum, suddenly became dilated and immovable.

The tongue had not fallen back upon the epiglottis; but it was pulled out nevertheless. The foot end of the table was elevated at once, while I induced artificial respiration by rhythmic compressions of chest and abdomen. After a few minutes the lividity of the face began to disappear, the pupil became movable and

---

*In the table of deaths from chloroform from 1869 to 1879, compiled by Dr. L. Turnbull, for the second edition of his work, "The Advantages and Accidents of Artificial Anæsthesia," I find that in only ten cases out to 160, death occurred *after* the anæsthesia.

contracted. When the patient began breathing and the foot end of the table was let down, the hiccoughing paroxysm returned, but was stopped by quickly elevating the board again. While the patient was still in this position I completed the second operation. Perhaps ten minutes later, the boy woke up and recovered without the least nausea.

*Remarks.*—In order to set this case in its proper light, I may state that the chloroform was kept in a cupboard with the door closed; that its purity was proven by the chemical tests; that the whole quantity gradually dropped upon the inhaler did not exceed thirty minims; that the apparatus was not applied again after I had commenced the operation; and that the patient lay perfectly horizontally during the narcosis. There was no organic lesion of the heart, no mechanical obstruction of the larynx; and since the boy did not vomit after the operation, I do not believe the asphyxia in this patient was due to food having been drawn up temporarily into the trachea by any effort at vomiting. I am rather inclined to believe that the asphyxia was due to the depressing and poisonous influence of the chloroform upon the nervous centers. The center of respiration became paralyzed, its regulatory influence upon the rhythmical movements of the muscles of respiration ceased, these movements became irregular and respiration spasmodic, hiccoughing. The defective respiration quickly loaded the blood with carbonic acid and thus produced a state of asphyxia which brought the patient to the verge of death.

In my own experience of fifteen years, this case is the first instance that a short and regular narcosis assumes a serious aspect at a time when the anæsthetic has been discontinued for a number of minutes and when we are accustomed to considering the patient safe and near his recovery. Dr. Laurence Turnbull, of Philadelphia, to whom I communicated this case, wrote to me that "the case of the boy was not so remarkable as to the time of the asphyxia; for on consulting my table of deaths from chloroform, which I had prepared for my second edition, I find there were ten deaths after operations, with chloroform poisoning mentioned as the cause." Still the case seems a little remarkable considering the small amount of the chloroform used and the briefness of its administration. For among the ten cases in Dr. Turnbull's list, there are only three which would compare with mine as to the short duration of the narcosis; in one case, chloroform was given for extracting a tooth; in the second case, for introducing a catheter; and in the third case, for the operation for fissure of the anus. In the other seven cases the amount of chloroform was larger (from two to twelve drachms) and the narcosis of a long duration (as for instance for amputation of thigh, parturition).

Dr. Turnbull also has kindly given me the notes of a case which is very similar to mine, but terminated fatally. A young girl, perhaps thirteen years, was operated upon by Professor Jaeger, of Vienna, for some trifling defect in her eye. The operation lasted but a few minutes, and was done under chloroform. A bandage was applied and the patient put to bed, already partly conscious. A few minutes later defective breathing with deep blueness of the skin was noticed. Agents for restoring consciousness were in vain employed; finally a tracheotomy was performed, and artificial respiration carried on by means of a bellows through the canula, but with no effect. Post mortem revealed nothing satisfactory.

